# The unique cognitive phenotype of ASD + ADHD co-occurrence: evidence for planning and attention deficits as a differentiating approach

**DOI:** 10.3389/fped.2025.1703264

**Published:** 2026-01-15

**Authors:** Tiantian Wang, Miaoshui Bai, Zunwei Zhang, Feiyong Jia

**Affiliations:** 1 Department of Developmental and Behavioral Pediatrics, Children's Medical Center, The First Hospital of Jilin University, Jilin University, Changchun, China; 2 Child Health Clinical Research Center of Jilin Province, Changchun, China

**Keywords:** attention-deficit/hyperactivity disorder, autism spectrum disorder, cognitive assessment system, cognitive function, co-occurrence

## Abstract

**Purpose:**

This study aimed to assess cognitive processing in children with autism spectrum disorder (ASD) without co-occurring attention-deficit/hyperactivity disorder (ADHD) (ASD-alone), children with ADHD without co-occurring ASD (ADHD-alone), and children with ASD with co-occurring ADHD (ASD + ADHD).

**Methods:**

Children were divided into four groups: ASD-alone (*n* = 57), ADHD-alone (*n* = 89), ASD + ADHD (*n* = 56), and typical development (TD) (*n* = 58). The Das–Naglieri Cognitive Assessment System (D-N CAS) was applied to evaluate planning, attention, simultaneous, and successive cognitive processes.

**Results:**

Children with ASD-alone scored less on planning processing. Children with ADHD-alone scored lower on planning and attention processing. Children with ASD + ADHD scored lower on all four processes. Planning and attention exhibited satisfactory stratification precision in identifying ASD + ADHD among children with ASD, with area under the curve (AUC) values of 0.7426 and 0.8061, respectively. Successive processing had medium diagnostic value in diagnosing ASD + ADHD among children with ADHD, with an AUC of 0.618. Inattention symptoms were associated with planning and attention processing. Social affects and inattention symptoms were associated with the total D-N CAS score.

**Conclusion:**

Children with ASD-alone, ADHD-alone, and ASD + ADHD exhibited distinct cognitive profiles. The D-N CAS, particularly its planning and attention scales, provided an approach for differential diagnosis in clinical settings.

## Introduction

Autism spectrum disorder (ASD) is characterized by abnormal social interactions, and restrictive repetitive behavioral patterns, and sensorimotor disorders. It has a prevalence of 2.7% (1 in 36) among children aged 8 years (1). Attention-deficit/hyperactivity disorder (ADHD) is characterized by inattentiveness and hyperactivity/impulsivity, with its prevalence varying across countries. Recent systematic reviews have indicated an overall global prevalence of approximately 8% in children (2, 3). ASD and ADHD are neurodevelopment disorders that have an enhanced co-occurrence rate. In particular, 32.8% of ASD children also have an ADHD co-occurrence, and 9.8% of children with ADHD have a co-occurrence of ASD (4). Moreover, individuals with co-occurring symptom presentations have more severe symptoms and greater functional impairment than children with ASD or ADHD-alone (5). Co-occurrence further exacerbates stress-, financial-, and time-related challenges within affected communities (6, 7).

Clinical diagnosis is complicated by co-occurrence. Several studies have shown that the presence of co-occurring ADHD is associated with a significant delay in ASD diagnosis (8). This is because ADHD symptomology often disguises ASD manifestations during clinical evaluations. This overlap may lead to greater challenges in identifying core ASD features during the diagnostic process (9). While this issue has garnered widespread attention, less emphasis has been placed on identifying ADHD when an ASD diagnosis has already been established. Clinically, both overdiagnosis of ADHD in individuals with ASD and missed diagnosis of ADHD are common (10, 11). This phenomenon is potentially attributed to the following factors (11–13): (a) Clinicians often struggle to distinguish whether the presentation of ADHD symptomatology stems from ASD manifestations. Yerys et al. found that existing ADHD rating scales do not precisely delineate inattentiveness and hyperactivity/impulsivity in ASD (11). Diagnostics relying on behavioral observations and parent-reported scales potentially lead to “false positives” in scale screenings, consequently misclassifying a portion of children with ASD without co-occurring ADHD (ASD-alone) as those having ASD co-occurring ADHD (ASD + ADHD). This misclassification may lead to overtreatment of ADHD—such as inappropriate use of stimulant medications—in children with ASD-alone. (b) “Search satisfying” refers to the termination of further assessments for secondary diagnoses once a diagnostician has made an ASD diagnosis (12). This missed diagnosis may impact long-term outcomes, as the child may miss out on timely early intervention (13). Studying ASD and ADHD in co-occurring patients is essential. In addition, the identification of robust screening and diagnostic tools for ASD + ADHD is critical.

Cognitive function is considered a prospective endophenotype of neurodevelopmental disorders (14). Most studies that have compared cognitive function between ADHD and ASD separately obtained conflicting results (15, 16). This inconsistency is likely because they did not assess co-occurring ADHD among ASD patient samples (17). Consequently, the inability to account for co-occurring ADHD in ASD-related investigations can lead to ambiguous conclusions (18). Therefore, further studies on ASD-alone, ADHD without co-occurring ASD (ADHD-alone), and ASD + ADHD are needed. Parallel studies have demonstrated that children with ASD + ADHD exhibit more cognitive impairments in attention, inhibition, and working memory (WM) than those with ASD-alone or ADHD-alone (19–22). However, there remain controversial perspectives regarding the core characteristics of the cognitive phenotype in ASD + ADHD. One group of studies supports the “additive impairment perspective,” with studies focusing on social cognition demonstrating that children with ASD + ADHD show exaggerated impairments, suggesting that their cognitive phenotype may be an accumulation of impairments from both disorders (14, 23, 24). The other body of research proposes the “unique phenotype perspective,” positing that the cognitive characteristics of ASD + ADHD are not merely the additive impairments of the two single disorders (25–30). Cremone-Caira et al. found that ASD + ADHD children exhibit a distinct pattern of reduced strategic response speed and inhibition of distracting information, differing from children diagnosed with either ASD-alone or ADHD-alone (26). Seernani et al. used oculomotor measures to demonstrate that ASD-specific weakened global processing involves different neural mechanisms in ASD + ADHD vs. ASD-alone. Their visual search data confirmed that ASD + ADHD cognitive traits are not a simple ASD-ADHD combination (28, 30). Notably, ASD + ADHD also differs in social cue processing, intra-subject variability, and facial memory, collectively confirming an independent neurocognitive phenotype (25, 29). The findings of different studies are heterogeneous, probably because they used different instruments and theoretical frames; moreover, these studies explored different cognitive domains. Although studying different cognitive domains is necessary, it is insufficient to understand the cognitive profiles of ASD + ADHD as these studies lack an exploration of general cognitive processes. In addition, these studies have not investigated the applicability of these assessment methods as diagnostic tools for ASD + ADHD.

The Planning, Attention, Simultaneous, Successive (PASS) theory is a well-defined theoretical framework (31). The theory redefines intelligence as a network of four cognitive processes: planning, attention, simultaneous, and successive processing. PASS theory proposed integrated cognitive processing in which attention processing operates under the overall monitoring of planning processing, while simultaneous and successive processing work in coordination. These four processes rely on three functional units within the brain (31). Planning processing involves determining, monitoring, and evaluating possible solutions to problems, and regulating actions to achieve the desired goal. It is primarily associated with the frontal lobe (third unit). Attention processing focuses on specific stimuli while disregarding competitive stimuli, and it utilizes the first unit—the brainstem and the midbrain. Simultaneous processing involves comprehension of overall information, while successive processing breaks down that information in a particular manner. These two processes reflect the execution of information encoding, which in the second unit—encompassing the occipital, temporal, and parietal lobes. PASS theory provides a multidimensional understanding of cognitive functioning to elucidate typical brain processing. This theory was comprehensive and could explain results employing other theoretical models (32). Planning and attention processing are strongly associated with executive functions (EF), including inhibition, updating, shifting, sustained attention, and selective attention (32). Simultaneous and successive processing are strongly correlated with visuospatial capacity and phonological abilities of WM (32). The Das–Naglieri Cognitive Assessment System (D-N CAS), derived from PASS theory, is a reliable and culture-free instrument for evaluating cognitive processing (33). Moreover, the D-N CAS has been standardized in China (34) and has been applied in studies evaluating the cognitive profiles of ADHD (35–38). In some studies, D-N CAS has also been investigated for its applicability as a diagnostic tool for ADHD (39, 40). Qin et al. found that the D-N CAS-based planning and attention processing evaluations exhibited enhanced sensitivity and specificity in ADHD diagnosis (40). Studies applying the D-N CAS to patients with ASD remain scarce. To date, only Taddei et al. have used D-N CAS in an ASD-related population to evaluate cognitive processing in children with ADHD and Asperger's syndrome (AS, a subtype of ASD). They found that AS children exhibited more severe impairments in planning and attention processing than those with ADHD (35). Notably, while D-N CAS has been relatively well utilized in ADHD research, its application in the broader ASD population (beyond AS) remains limited, and no studies have specifically evaluated cognitive processing profiles in children with ASD + ADHD using this tool.

To better identify neurocognitive endophenotypes of ASD + ADHD, we analyzed the cognitive processing in this population using the D-N CAS. This study aimed to (a) compare cognitive processing among children with ASD-alone, ADHD-alone, and ASD + ADHD; (b) assess the diagnostic performance of D-N CAS in Chinese children with ASD + ADHD; and (c) analyze the relations between the clinical symptoms and the standard scores of D-N CAS. Based on previous investigations into the cognitive profiles in ASD and ADHD, we formulated the following hypothesis: (1) Children in the ASD-alone, ADHD-alone, and ASD + ADHD groups will exhibit distinct cognitive processing profiles. The cognitive profiles of children with ASD + ADHD are not accumulative impairments of pure ASD or pure ADHD. (2) D-N CAS assessment will demonstrate diagnostic value for ASD + ADHD. (3) D-N CAS scores will be associated with clinical symptoms.

## Methods

### Participants

Our protocols received ethical approval from the First Hospital of Jilin University. Data were collected from children diagnosed with ASD or ADHD who visited the outpatient clinic of the Department of Developmental and Behavioral Pediatrics. These children were classified into three groups: ASD + ADHD, ASD-alone, and ADHD-alone. In addition, we collected medical information from neurotypical children who visited the clinic for routine check-ups; these children constituted the typical development (TD) group. All children with suspected ASD or ADHD underwent a systematic and rigorous clinical evaluation. This evaluation included direct structured interviews with the participants and their caregivers, standardized behavioral observations, and a comprehensive review of medical records and developmental histories. Diagnoses were confirmed by a multidisciplinary team consisting of child and adolescent psychiatrists, developmental behavioral pediatricians, and clinical psychologists—all trained in neurodevelopmental disorder assessment. The diagnostic process strictly adhered to the criteria outlined in the American Psychiatric Association's Diagnostic and Statistical Manual of Mental Disorders, 5th Edition (DSM-5), ensuring clinically grounded and standardized assessment. For children initially diagnosed with ASD, co-occurring ADHD was diagnosed via a two-step evaluation: (1) screening with the Vanderbilt ADHD Diagnostic Parent Rating Scale (41); (2) confirmation by the multidisciplinary team using DSM-5 criteria, incorporating behavioral observations, caregiver interviews, and review of developmental histories to distinguish ADHD-specific symptoms from ASD-related manifestations. ADHD subtypes (inattentive, hyperactive-impulsive, combined) were categorized based on the Vanderbilt ADHD Diagnostic Parent Rating Scale (41). Autism severity was assessed using the Autism Diagnostic Observation Schedule-Second Edition (ADOS-2), with severity levels determined by calibrated severity scores (42). Children in the ADHD-alone and TD groups underwent screening for ASD using the original Social Responsiveness Scale (SRS)—a validated screening tool for ASD in pediatric populations (43). A cutoff SRS Total T-score >65 was applied, which is consistent with established criteria for identifying individuals at high risk of ASD. All participants were required to have an intelligence quotient (IQ) ≥80, validated by the Wechsler intelligence scale for children, as revised in China, to ensure that cognitive profiles were not due to intellectual disability (44). Exclusion criteria included taking any active central nervous system medication, other co-occurring psychiatric disorders, and a diagnosis of epilepsy.

A total of 260 subjects aged 5–16 years were included in the study. Of these, 89 were categorized into the ADHD-alone group, 57 into the ASD-alone group, and 56 into the ASD + ADHD group. Fifty-eight participants without any physical or mental diseases were enrolled as TD control. Sex did not differ significantly by groups (χ^2^ = 7.293; *P* = 0.063). Age differed significantly by groups (*H* = 29.277, *P* < 0.0001). Based on *post hoc* analyses, the ages of the children in the TD and ADHD-alone groups were substantially higher, compared to the ASD-alone children. The children in the ADHD-alone group were substantially older relative to the ASD + ADHD children. Total T-score of SRS differed significantly by groups (*F* = 153.58, *P* < 0.0001). The ASD-alone and ASD + ADHD groups scored higher compared with the ADHD-alone group. The ADHD children scored higher relative to the TD children. No significant alteration was observed in IQ between the groups (*F* = 6.100, *P* = 0.107). The demographic and clinical characteristics of all participants are summarized in Table 1.

**Table 1:** Clinical and demographic features of the sample.

Variables	1. ASD-alone (*n* = 57)	2. ASD + ADHD (*n* = 56)	3. ADHD-alone (*n* = 89)	4. TD (*n* = 58)	*F/H/*χ^2^	*P*	*Post hoc*
Age (years)	6 (5.5, 8)	7 (6, 8)	9 (7, 11)	8 (6, 11.25)	29.28	<0.0001	1 < 3, 4*; 2 < 3*
Gender (male)	44 (77.2%)	52 (92.9%)	75 (84.3%)	44 (75.9%)	7.29	0.06	–
IQ	98 (85.5, 109)	96 (93, 102)	97 (93, 102.5)	101.5 (96, 106)	6.10	0.11	–
ADHD subtype					4.74	0.09	–
Combined	–	23 (41.1%)	53 (59.6%)	–	–	–	–
Inattentive	–	4 (7.1%)	4 (4.5%)	–	–	–	–
Hyperactive-impulsive	–	29 (51.8%)	32 (36%)	–	–	–	–
ASD severity					1.03	0.60	–
Mild	33 (57.9%)	25 (44.6%)	–	–	–	–	–
Moderate	15 (26.3%)	26 (46.4%)	–	–	–	–	–
Severe	9 (15.8%)	5 (8.9%)	–	–	–	–	–
SRS	71.64 ± 1.64	75.47 ± 1.63	38.79 ± 1.30	32.34 ± 1.60	153.58	<0.0001	1,2 > 3 > 4*
ADOS SA	7 (6, 8)	7 (7, 8.75)	–	–	−1.51	0.13	
ADOS RRB	1 (0, 1)	0 (0, 1.75)	–	–	−0.56	0.57	

TD, typically developing controls; ASD-alone, autism spectrum disorder without co-occurring attention deficit hyperactivity disorder; ADHD-alone, attention deficit hyperactivity disorder without co-occurring ASD; ASD + ADHD, co-occurring ASD and ADHD; IQ, intelligence quotient; SA, social affect; RRB, restricted, repetitive behaviors; ADOS-2, autism diagnostic observation schedule-second edition module; SRS, social responsiveness scale.

*Post hoc test *p* < 0.05.

### Measures

Well-trained psychotherapists, blinded to the participants' diagnoses, administered the Chinese version of the D-N CAS in this study. The D-N CAS has four sections: planning, attention, simultaneous, and successive processing. Each scale contains three subtests. Therefore, 12 subtests were performed for the D-N CAS test. It takes approximately 60–90 min for a single subject to complete all tests. The Chinese version of D-N CAS has demonstrated good reliability (Cronbach's *α* = 0.70–0.89 for subscales; test–retest reliability *r* = 0.72–0.90) (34).

#### Planning processing

Planning processing assessment was used to evaluate the decisions taken, strategies selected, and solutions identified for problems, for example, matching numbers, planned codes, and planned connections. Regarding matching numbers, the child was asked to select two identical numbers in each row, with number arrangements designed to detect the matching strategy. The planned code subtest consisted of two pages containing independent code sets and specific arrangements. The letter codes were listed at the top of each page. Children were asked to place the given code in an empty space under the letter, in whatever fashion they desired. Regarding planned connections, children were asked to randomly link scattered numbers and letters in a sequential manner (e.g., 1-2-3-4/1-A-2-B-3-C).

#### Attention processing

Attention processing assessment was used to evaluate selective focus on specific stimuli while dismissing other stimuli, such as expressive attention, number detection, and receptive attention. The expressive attention subtest measures a child's performance in inhibiting interfering stimuli. Children were presented with words printed in color, with the words and colors of the font being incongruent. Children were required to identify a word color rather than the word itself. Number detection assesses selectivity, attention division, and distraction resistance. Children were required to identify numbers that looked the same as those listed in an example. Children were instructed against skipping a page. Receptive attention requires a child to find every pair of pictures or letters with similar or consistent characteristics.

#### Simultaneous processing

Simultaneous processing requires integration of several stimuli into a perceptual or conceptual whole. It consists of nonverbal matrices, verbal-spatial relationships, and figure memory. The nonverbal matrices subtest involves shapes and geometric patterns interconnected through spatial or logical arrangement. Children were asked to deduce associations between item components and identify the missing figure out of six possible answers. The verbal-spatial relationships subtest requires children listen to logical and grammatical accounts of spatial associations and choose a picture conforming with the verbal account. In the figure memory subtest, a child was provided with a two- or three-dimensional geometric figure, which was removed 5 s later; the child was then instructed to find the original figure, which was integrated within a larger and more intricate geometric pattern.

#### Successive processing

Successive processing was used to evaluate the mechanism whereby a child processes stimuli in a particular manner. It consists of word series, sentence repetition, and sentence questions. In word series, the child was required to pay attention to a string of one-syllable words read by the administrator at 1 word per second. Subsequently, the child was asked to repeat the words in the same sequence. Individual words series ranged between two and nine words. In sentence repetition, the child was required to repeat the auditory sentence, which was composed of color words and nonsense words. In the sentence questions, children aged 8–17 years were instructed to listen to a sentence and then answer a question about the sentence. In the speech rate subtest, children aged between five and seven years were asked to repeat one- and two-syllable words ten times as soon as possible. The raw scores of the 12 subtests were calculated separately and then converted into scale scores. The scale scores were summed for each subscale and then transformed into standard scores.

### Statistical procedures

Data analysis was performed using SPSS 26.0 software. Continuous data displaying non-normal distributions are presented as medians (25th–75th percentiles). We used a Kruskal–Wallis test for independent samples to analyze group differences when the variables were non-normally distributed variables, and Bonferroni for *post hoc* comparison. Categorical data are represented as counts and percentages. For inter-independent cohort analyses, we employed the χ^2^ test or Fisher's exact test when necessary. Continuous data displaying normal distributions are presented as mean ± standard deviation (SD). Due to significant group differences in age, we employed univariate analyses of covariance (ANCOVAs) for inter-group comparisons with age as a continuous covariate. Dependent data were the standard scores of D-N CAS. Bonferroni *post hoc* analyses were performed for inter-group analyses, when a significantly group effect was identified.

We performed ROC analysis to evaluate the diagnostic value of D-N CAS in identifying ASD co-occurring ADHD among children with ASD or ADHD. A threshold of *P* < 0.05 was considered statistically significant.

Multiple linear regression analysis was performed to analyze the relations between the clinical symptoms and the standard scores of D-N CAS. Bonferroni correction was applied to control for Type I error in multiple linear regression analyses. The standard score of each section of D-N CAS was the dependent variable. Gender, age, ADHD symptoms (inattention and hyperactivity subscales of Vanderbilt Scale), and ASD features [social affect (SA) and restricted, repetitive behaviors (RRB) subscales of Autism Diagnostic Observation Schedule-Second Edition Module (ADOS-2)] were independent variables (41, 42).

## Results

### D-N CAS-based assessment of ASD-alone, ASD + ADHD, ADHD-alone, and TD children

The ANCOVAs revealed significant differences between groups on planning processing, *F* = 48.419, *P* < 0.0001; simultaneous processing, *F* = 5.509, *P* = 0.001; attention processing, *F* = 59.197, *P* < 0.0001; successive processing, *F* = 4.438, *P* = 0.005; and full scale, *F* = 33.975, *P* < 0.0001. According to our *post hoc* analyses, there were significant differences between the TD and ASD + ADHD groups on all analyzed D-N CAS variables. The ASD + ADHD group produced significantly lower scores, compared with the TD group on planning, simultaneous, attention, successive processing and full scale. *Post hoc* analyses highlighted significant differences between the TD and ASD-alone groups on planning processing and the full scale of the D-N CAS. There were no significant differences on simultaneous, attention, and successive processing. The ASD-alone group showed a significantly lower score than the TD group on planning processing and full scale. *Post hoc* analyses demonstrated significant differences between the TD and ADHD-alone groups on planning, attention, and full scale of the D-N CAS. Conversely, no significant differences were present among simultaneous and successive processing. The ADHD-alone group produced significantly lower scores than the TD group on planning, attention processing, and full scale. The ASD-alone group scored significantly higher than the two groups (ADHD-alone and ASD + ADHD) on planning, attention processing, and full scale. Meanwhile, the ADHD-alone group scored substantially more, relative to the ASD + ADHD group, on successive processing (Table 2).

**Table 2:** Differences between groups on D-N CAS.

D-N CAS	1. ASD-alone (*n* = 57)	2. ASD + ADHD (*n* = 56)	3. ADHD-alone (*n* = 89)	4. TD (*n* = 58)	*F*	*P*	*Post hoc* Bonferroni
Planning	88.00 ± 13.06	76.43 ± 11.87	77.88 ± 9.28	95.72 ± 6.85	48.419	<0.0001	3, 2 < 1 < 4*
Simultaneous	115.16 ± 18.33	111.23 ± 14.74	115.19 ± 11.76	121.40 ± 11.01	5.509	0.001	2 < 4*
Attention	97.11 ± 11.73	84.05 ± 10.23	82.69 ± 9.27	101.57 ± 8.15	59.197	<0.0001	3, 2 < 1, 4*
Successive	107.53 ± 12.21	103.93 ± 12.00	108.64 ± 8.98	110.03 ± 9.21	4.438	0.005	2 < 3, 4*
Full scale	102.32 ± 13.18	91.54 ± 12.64	94.64 ± 9.38	109.12 ± 6.32	33.975	<0.0001	2, 3 < 1 < 4*

TD, typically developing controls; ASD-alone, autism spectrum disorder without co-occurring attention deficit hyperactivity disorder; ADHD-alone, attention deficit hyperactivity disorder without co-occurring ASD; ASD + ADHD, co-occurring ASD and ADHD.

* *Post hoc* test *p* < 0.05.

### ROC analyses

#### D-N CAS-based ASD + ADHD diagnostic performance among ASD children

**Figure 1:**
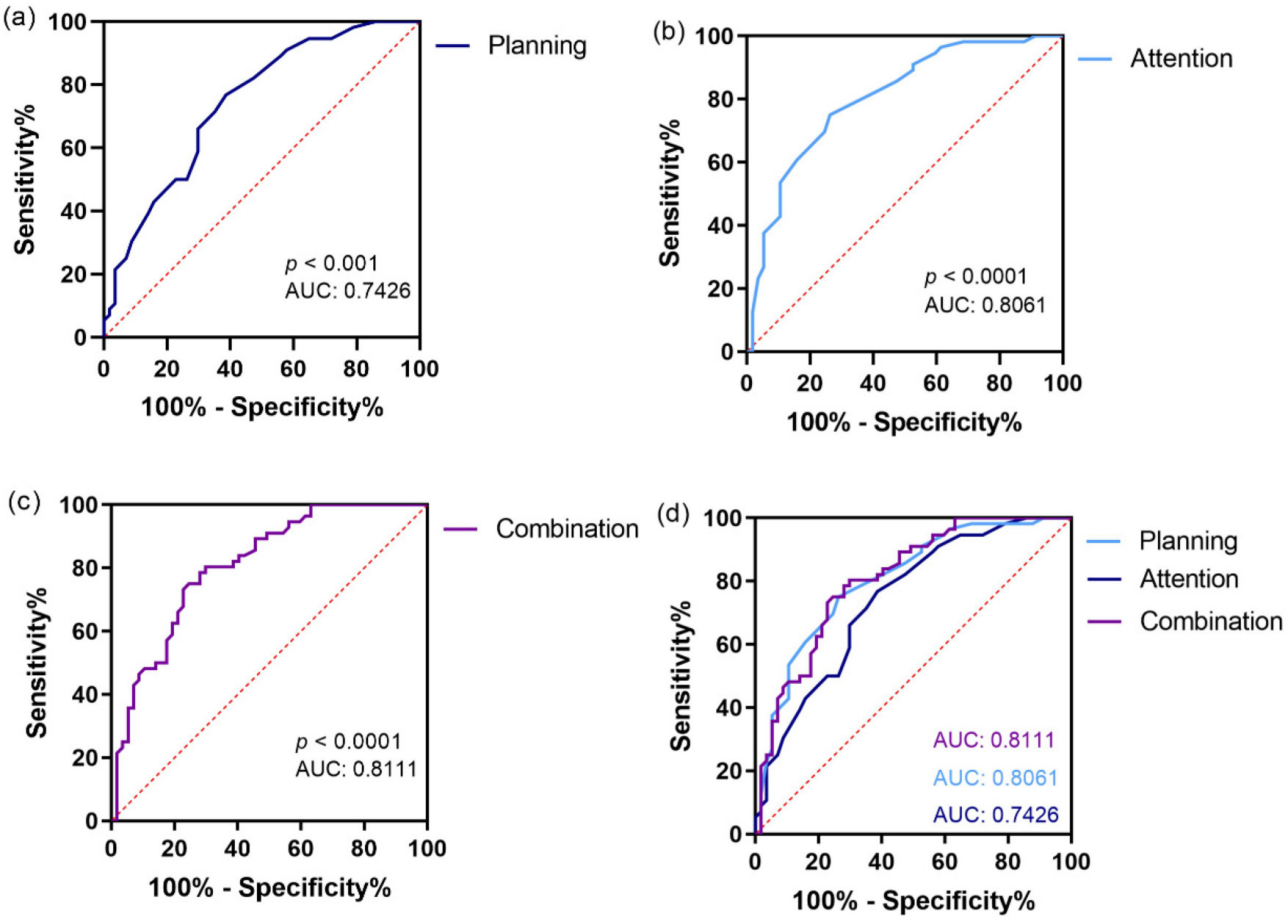
The ROC curve of planning, attention, and their combination in diagnosing ASD + ADHD in children with ASD. **(a)** The ROC curve for planning in diagnosing ASD + ADHD in children with ASD. **(b)** The ROC curve for attention in diagnosing ASD + ADHD in children with ASD. **(c)** The ROC curve for planning and attention in diagnosing ASD + ADHD in children with ASD. **(d)** The total ROC curve of planning, attention, and their combination. AUC, area under the curve.

The aforementioned results demonstrated that planning and attention processing were significantly different between the ASD + ADHD and ASD-alone groups. Therefore, we investigated the diagnostic value of planning and attention processing evaluation. Based on our ROC analysis, planning demonstrated satisfactory stratification precision for ASD + ADHD diagnosis with area under the curve (AUC) of 0.7426 (95% CI: 0.6526–0.8327, *P* < 0.0001). The cutoff point for planning standard score was 86. The planning processing yielded a sensitivity of 61.4% and a specificity of 76.8%. Attention evaluation revealed satisfactory stratification precision for ASD + ADHD diagnosis with AUC of 0.8061 (95% CI: 0.7260–0.8861, *P* < 0.0001). The cutoff point for attention standard score was 91. The attention processing yielded a sensitivity of 73.7% and a specificity of 75%. We demonstrated that integration of planning and attention processing produced the optimal diagnostic performance with an AUC of 0.8111 (95% CI: 0.7328–0.8894, *P* < 0.0001). In fact, it produced the highest sensitivity [sensitivity: combination (75%) vs. planning (61.4%) vs. attention (73.7%)]. However, the specificity of the planning and attention processing integration was significantly reduced compared with the planning processing alone [specificity: combination (75.4%) vs. planning (76.8%)] (Figure 1).

#### D-N CAS-based ASD + ADHD diagnostic performance among ADHD children

**Figure 2:**
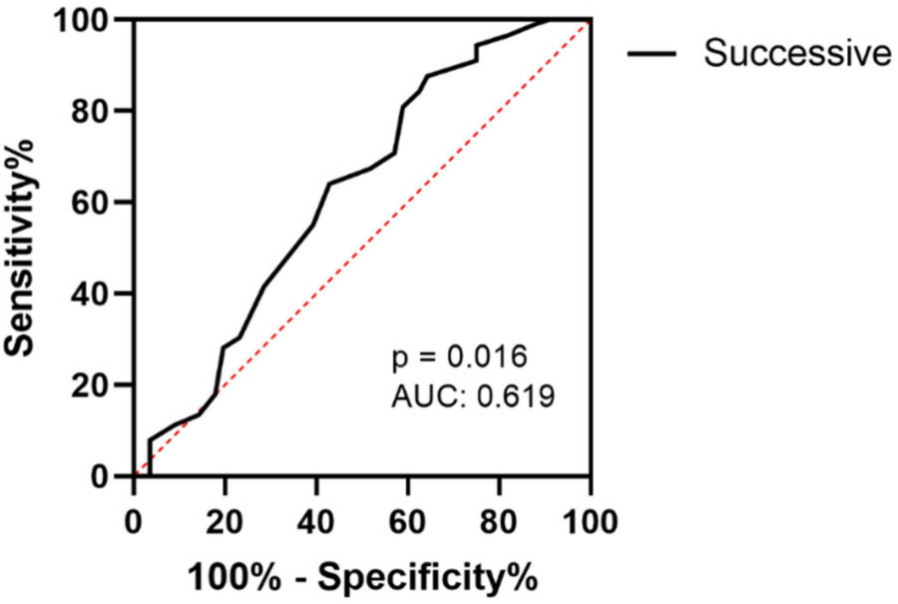
The ROC curve of successive processing in diagnosing ASD + ADHD in children with ADHD.

Successive processing was found to significantly different between the ASD + ADHD group and the ADHD-alone group. Therefore, we investigated the diagnosis value of successive processing assessment. The ROC analysis indicated that successive processing had medium stratification precision among the ASD + ADHD group diagnosed with AUCs of 0.618 (95% CI: 0.522–0.717, *P* = 0.016). The cutoff point for successive processing standard score was 103. Successive processing yielded a sensitivity of 70.8% and a specificity of 42.9% (Figure 2).

### Relations between ADHD/ASD features and the D-N CAS

**Figure 3:**
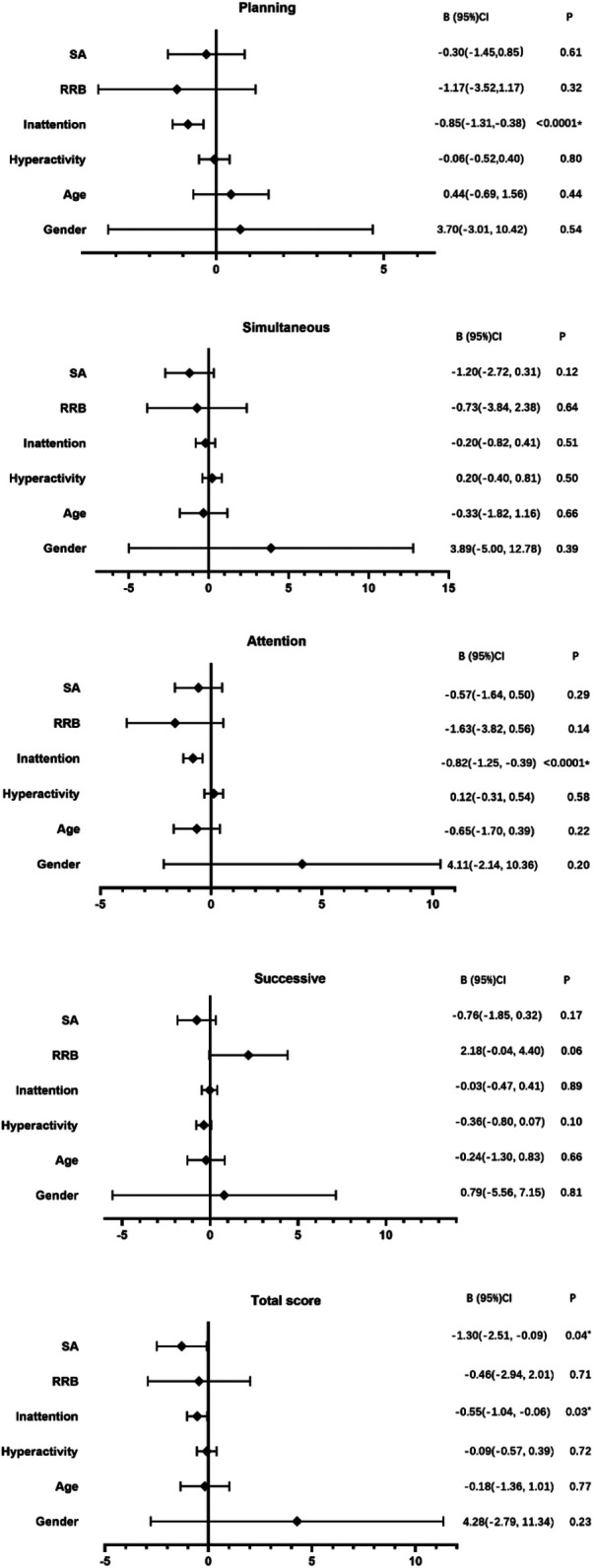
Relations between ADHD/ASD features and the D-N CAS.

Inattention symptoms were strongly related to poorer performance on planning processing and attention processing (*B* = −0.85, *P* < 0.0001, *B* = −0.82, *P* < 0.0001, respectively). Poor SA and inattention symptoms were related to lower total scores of D-N CAS. No significant associations were observed between clinical symptoms and performance on simultaneous and successive processing, though the results demonstrated the trend that the higher the RRB score of ADOS, the higher the successive processing score (*B* = 2.18, *P* = 0.06) (Figure 3).

## Discussion

In this study, we investigated the profiles of cognitive processing evaluated by D-N CAS among children with ASD-alone, ADHD-alone, ASD + ADHD, and TD. This study also evaluated the diagnostic performance of the D-N CAS in Chinese children with ASD + ADHD.

Our study observed a male-to-female ratio in the combined cohort of ASD-alone and ASD + ADHD that was slightly higher than the widely reported ratio in pediatric ASD populations (1). The ASD-alone group's gender ratio aligned with widely reported ratio, while the ASD + ADHD group showed elevated male predominance. This may reflect the inherent male bias of ADHD (2), compounded by the “additive effect” of ASD and ADHD gender biases, as well as diagnostic factors (e.g., females with isolated ASD “camouflage” symptoms, while co-morbid ADHD amplifies behavioral visibility in males) (45). Our findings highlight the need for comorbidity stratification in analyzing neurodevelopmental disorder gender characteristics, particularly vigilance for underdiagnosed ASD in females without ADHD.

As hypothesized, children with ASD-alone, ADHD-alone, and ASD + ADHD demonstrated different profiles of cognitive processing. Children with ASD + ADHD showed deficits across all four processes. Children with ASD-alone demonstrated impairments in planning processing; their performance in attention, simultaneous, and successive processing, however, was comparable to that of TD children. For children with ADHD-alone, deficits were concentrated in planning and attention processing; their simultaneous and successive processing abilities, by contrast, were on par with those of the TD group. Furthermore, our results revealed that children with ASD + ADHD exhibited more severe impairments in attention and planning than those with ASD-alone, and poorer successive processing performance than those with ADHD-alone. Our analysis suggested that co-occurrence is partially attributed to the cumulative effects of the cognitive deficits associated with pure ASD and pure ADHD. Notably, ASD + ADHD also presented unique cognitive indicators, implying a distinct clinical profile. Distinct processing or other factors might underlie the cognitive outcome in co-occurring patients. Our research provides preliminary evidence that different deficit combinations of the four processes of PASS may help differentiate ASD-alone, ADHD-alone, and ASD + ADHD from controls in clinical practice.

Our findings for ADHD-alone were consistent with prior studies regarding the cognitive profiles of individuals with ADHD evaluated via D-N CAS. A majority of prior investigations revealed that children with ADHD exhibit deficits in planning and attention processing, yet achieve average scores on the simultaneous and successive scales of D-N CAS (32, 37). Furthermore, in our results, ADHD as a whole (ASD + ADHD group, ADHD-alone group) demonstrated deficits in planning and attention processing. This observation provides additional support for the proposition that attention and planning deficits represent a potential diagnostic phenotype for ADHD. Qin et al. reported that children with ADHD exhibited impairments in simultaneous process alongside deficits in planning and attention (40). A plausible explanation for this discrepancy is that the ADHD sample in that study may have included children with undiagnosed ASD + ADHD. We found that the cognitive profiles of ASD-alone patients failed in planning processing and were normal in attention, simultaneous, and successive processing. These characteristics differ from those reported by Taddei et al. who identified both planning and attention deficits in children with ASD (35). However, whether the children with ASD in their study had ADHD remains unclear, and this ambiguity likely accounts for the divergent findings between the two studies. Our findings also highlight the relevance of routine ADHD co-occurrence consideration during ASD assessment to avoid misleading conclusions. ASD co-occurrence should also be considered to prevent misleading results when studying ADHD. Distinguishing co-occurrence is necessary, which may provide ideas for intervention.

To our knowledge, no prior study has utilized the D-N CAS to assess cognitive processing profiles in children with ASD + ADHD, though Sinzig et al. reported more severe impairments in alertness, inhibition, flexibility, attention, and planning in ASD + ADHD children via other tasks (20, 22, 26, 46). Our results showed that children with ASD + ADHD exhibited deficits in attention and planning processing, consistent with aforementioned studies. Naglieri et al. emphasized that planning and attention processing assessed by the D-N CAS represented a complex measure of EF (47). Planning processing was linked to updating, inhibition, and shifting of EF (46, 47). The three subtests of attention processing in D-N CAS were linked to sustained attention and selective attention of EF. The cognitive processing profile in this study can explain previous investigation conclusions from different tasks. However, our data also suggest deficits in simultaneous and successive processing. Takeuchi et al. found that children with ASD + ADHD had deficits in verbal WM and visuospatial WM, assessed by the digit span and visuospatial span tasks, respectively (21). The digit span task and visuospatial span tasks are similar to the word series subtest and figure memory subtest of the D-N CAS, supporting the relevance of PASS theory in partly explaining WM.

As hypothesized, our ROC analysis results provided evidence that attention and planning processing can serve as effective and objective biomarkers to assist in clinical differential diagnosis. Both domains showed meaningful discriminatory power: Attention had a higher AUC (0.8061) than planning (0.7426), while their composite indicator achieved performance (AUC = 0.8111) with significantly enhanced sensitivity. This finding carries significant clinical implications. Inattention, hyperactivity, and impulsivity behaviors in children with ASD are heterogeneous: They may stem from co-occurring ADHD or reflect core ASD features (e.g., poor social motivation, sensory issues) rather than independent ADHD. Behavioral observations and parent-reported scales risk false positives and misclassify a portion of ASD-alone children as ASD + ADHD. Our findings offer a new perspective for addressing this clinical dilemma. The objective assessment of D-N CAS can more directly tap into the core cognitive deficits of ADHD. For children with ASD with hyperactive/inattentive behaviors, significant impairments in attention and planning of D-N CAS strongly indicate that these behaviors originate from co-occurring ADHD. Conversely, near normal cognitive assessment suggests that their behaviors are more likely attributable to ASD itself. This differentiation has direct implications for intervention guidance. For those with true co-occurrence, ADHD treatment, such as methylphenidate hydrochloride, may effectively alleviate core symptoms. In contrast, for children whose behaviors are due to ASD itself, the use of such medications is often ineffective and may increase the risk of side effects such as insomnia, decreased appetite, and mood fluctuations. Thus, the D-N CAS can help clinicians make safer and more effective interventions.

Furthermore, this study also explored the discriminative value of the D-N CAS from another perspective: distinguishing ASD + ADHD among those diagnosed with ADHD. ROC analysis revealed that the AUC of successive processing was 0.618. However, its accuracy was lower than the efficacy of planning and attention in discriminating ADHD within children with ASD. This finding revealed an asymmetry in co-occurrence diagnosis. It suggested that using the D-N CAS to identify ASD in ADHD may be more challenging than identifying ADHD in ASD. Nevertheless, the relatively high sensitivity (70.8%) suggested that significant impairment in successive processing may serve as an effective “screening signal” to alert clinicians to the potential possibility of co-occurring ASD in children with ADHD. However, it is not sufficient as a definitive diagnostic tool. Accurate identification must involve a comprehensive assessment that integrates core symptomatic dimensions of ASD, such as social communication deficits and restricted, repetitive behaviors.

Beyond their diagnostic utility, our findings suggested that inattentive symptoms may be related to poorer attention and planning processes. This was similar to previous studies on EF (20). These correlational analyses mechanistically explain our ROC results by linking planning/attention processing to ADHD's core inattentive phenotype, confirming that these cognitive domains are not merely laboratory measures but foundational to ADHD symptom expression in ASD children. In addition, we also found that worse SA symptoms were related to poorer total score of D-N CAS, indicating that social impairments may be associated with total cognitive processing. However, no significant relationships were observed between ASD features and each cognitive processing domain of PASS. Although restrictive and repetitive behaviors (RRB) symptoms of ASD showed a potential association with successive processing, it did not reach statistical significance, possibly due to the limited sample size. Successive processing involves sequential and temporal information processing, a skill that may be enhanced in some individuals with ASD who exhibit a strong preference for patterns, routines, and systematic details. Further studies with large sample sizes focused on cognitive processes and ASD features are required.

## Limitations

This study has some limitations. First, the sample size was relatively small, which constrains the statistical reliability of the findings. Second, the study groups were not homogeneous in terms of age and gender. Although we performed statistical adjustment to mitigate potential confounding effect, inherent demographic heterogeneity may still restrict the external validity of the results, limiting their generalizability to broader populations. Future studies should expand the sample size and ensure homogeneity across age and gender. Such efforts will help validate the current findings and enhance the robustness of conclusions drawn from this study.

## Conclusion

Our data revealed that children with ASD-alone, ADHD-alone, and ASD + ADHD exhibited different profiles of cognitive processing compared with the TD group. The D-N CAS differentiated ASD-alone, ADHD-alone, and ASD + ADHD patients from controls. Co-occurrence could be partially caused by an accumulative co-emergence of pure ASD and ADHD, but with certain unique cognitive profiles. The D-N CAS may be a useful instrument for differential diagnosis. It can help circumvent the limitations of behavioral questionnaires, reduce misdiagnosis, and inform more precise intervention strategies for children with complex neurodevelopmental presentations. Further studies using D-N CAS with larger sample sizes should be conducted to enable clinicians to better understand the characteristics of ASD + ADHD children to make accurate diagnosis and administer effective pharmacological and non-pharmacological treatment.

## Data Availability

The original contributions presented in the study are included in the article, further inquiries can be directed to the corresponding author.
